# HPLC Analysis of Phenolics Compounds and Antioxidant Capacity of Leaves of *Vitex megapotamica* (Sprengel) Moldenke

**DOI:** 10.3390/molecules18078342

**Published:** 2013-07-16

**Authors:** Thiele Faccim de Brum, Marina Zadra, Mariana Piana, Aline Augusti Boligon, Janaina Kieling Fröhlich, Robson Borba de Freitas, Sílvio Terra Stefanello, Amanda Luana Forbrig Froeder, Bianca Vargas Belke, Letícia Teixeira Nunes, Roberta da Silva Jesus, Michel Mansur Machado, João Batista Teixeira da Rocha, Félix Alexandre Antunes Soares, Margareth Linde Athayde

**Affiliations:** 1 Post-Graduate Program in Pharmaceutical Sciences, Federal University of Santa Maria, Camobi Campus, Santa Maria, RS, 97105-900, Brazil; 2 Post-Graduate Program in Biochemical Toxicology, Federal University of Santa Maria, Camobi Campus, Santa Maria, RS, 97105-900, Brazil; 3 Pharmacy Course, Federal University of Santa Maria, Camobi Campus, Santa Maria, RS 97105-900, Brazil; 4 Post-Graduate Program in Pharmaceutical Sciences, Federal University of Pampa-UNIPAMPA, Uruguaiana, RS 97500-970, Brazil

**Keywords:** *Vitex megapotamica*, phenolic compounds, antioxidant capacity, HPLC/DAD

## Abstract

*Vitex megapotamica* (Sprengel) Moldenke belongs to the Verbenaceae family and is popularly known as “tarumã”. The antioxidant capacity of fractions and crude extract from the leaves of *V. megapotamica* were determined in this study through the capacity to remove reactive species and phenolic compounds were quantified in the various fractions. The IC_50_ (DPPH) ranged from 14.17 ± 0.76 to 37.63 ± 0.98 µg/mL. The ethyl acetate fraction might contain the strongest lipid peroxidation inhibitory compounds with an IC_50_ of 16.36 ± 5.09 µg/mL, being also the one with the highest content of polyphenols (522.4 ± 1.12 mg/g), flavonoids (220.48 ± 0.30 mg/g) and condensed tannins (3.86 ± 0.53 mg/g). Compounds quantified by HPLC/DAD in the crude extract and fractions were chlorogenic and rosmarinic acids. Higher dosages of the extracts were more effective in reducing levels of plasma protein carbonyls and were also shown to be able to remove reactive species by a 2',7'-dichlorofluorescein diacetate assay, reducing oxidative stress in all tested fractions. Results obtained indicated that *V. megapotamica* exhibits good potential to prevent diseases caused by the overproduction of free radicals and it might also be used as a potential source of natural antioxidant agents.

## 1. Introduction

Numerous crude extracts and pure natural compounds from plants are reported to have antioxidant and radical-scavenging activities and intensive research has been carried out, either to characterize the antioxidant properties of extracts and/or to isolate and identify the compounds responsible for those activities seeking the development of natural antioxidant formulations in the areas of food, medicine and cosmetics [1,2,3,4].

Phenolic compounds are known to be responsible for the free radical scavenging and antioxidant activities of plants; they possess many biological effects, mainly attributed to their antioxidant activities in scavenging free radicals, inhibition of peroxidation and chelating transition metals. Free radicals are reactive oxygen species (ROS), which include hydrogen peroxide, hydroxyl radical, nitric oxide, peroxynitrite, singlet oxygen, peroxyl radical, and superoxide anion [5]. An over-production of these reactive species will result in oxidative stress brought about by the imbalance of the bodily antioxidant defense system and free radical formation. Oxidative stress has been shown to be one of the mechanisms that correlates with oxidative damages caused by free radicals and leading to chronic diseases like cancer, coronary heart diseases, diabetes, neurodegenerative diseases, and even aging [6]. Different therapeutic strategies have been proposed for the prevention and treatment of ROS-mediated diseases, with special emphasis on antioxidant therapy, that can delay or inhibit the oxidation of lipids or other molecules by inhibiting the initiation or propagation of oxidative chain reactions.

*Vitex megapotamica* (Sprengel) Moldenke belongs to the Verbenaceae family and is popularly known as “tarumã”. It is found distributed in northeastern Argentina, eastern Paraguay, in Uruguay and is commonly found in southern Brazil. In traditional medicine, the infusion of the leaves of this plant is used as a diuretic and hypocholesterolemic, as anti-inflammatory, in cases of rheumatism, treatment of hemorrhoids and skin disorders, among other uses [7]. Older studies of *V. megapotamica* have reported the isolation of pterosteron, polypodin B, phytoecdysones and ecdysonatiges steroids and iridoids [8,9]. A previous study demonstrated that *V. megapotamica* has an anti-hyperglycemic action and is able to improve the diabetic state, being probably a source of hypoglycemic compounds [10]. The hypolipidemic effect of *V. megapotamica* was also determined, showing a reduction of serum cholesterol and triacylglycerol [11]. A phytochemical screening of tarumã revealed the presence of anthocyanin glycosides, tannins, catechins, flavonols, xanthones, triterpenoid steroids, cardioactive glycosides, coumarins and organic acids [12]. Recently, the chemical composition of the essential oil of *V. megapotamica* leaves was verified, proving their antioxidant activity [13]. Therefore, this study aimed to quantify total polyphenols, flavonoids and tannins in the crude extract and dichloromethane, ethyl acetate and n-butanol fractions from leaves of *V. megapotamica.* Additionally, rosmarinic (RA) and chlorogenic (CLA) acids were quantified by high performance liquid chromatography with diode array detection (HPLC-DAD). The antioxidant capacity was evaluated by 1,1-diphenyl-2-picrylhydrazyl (DPPH), inhibition of lipid peroxidation (TBARS) and protein oxidation (carbonyl groups) assays. In addition, the ROS scavenging capacity was evaluated by using the 2',7'-dichlorofluorescein diacetate (DCFH-DA) oxidation test.

## 2. Results and Discussion

### 2.1. Phenolics Contents, Total Flavonoids, Condensed Tannins and Free Radical-Scavenging Activities

Studies of the qualitative composition of plant extracts revealed the presence of high concentrations of phenolics in the extracts obtained using polar solvents. Various phytochemical components, especially polyphenols, are known to be responsible for the free radical scavenging and antioxidant activities of plants [14]. Total phenolic (TP), total flavonoids (TF), condensed tannins (CT) and DPPH (IC_50_) values are given in Table 1. The ethyl acetate fraction was the one with the highest content of polyphenols and flavonoids in this study [522.4 mg/g gallic acid equivalents (GAE) and 220.48 mg/g of rutin equivalents (RE), respectively]. High contents of polyphenols and flavonoids in the ethyl acetate fractions were also reported by Boligon *et al*. [15] working with *Scutia buxifolia* leaves (322.69 and 145.72 mg/g phenolics and flavonoids, respectively), Janovik *et al*. [16], studying *Cariniana domestica* leaves (510.00 and 39.92 mg/g) and Schubert *et al*. [17], working with *Ilex paraguariensis* leaves and fruits whose phenolic contents ranged from 86.82 to 199.91 mg/g.

**Table 1:** Total polyphenols, total flavonoids, condensed tannins and antioxidant capacity (IC_50_/DPPH) for crude extract and fractions of *V. megapotamica*.

Extract/fraction	TP ± SD (mg GAE/g)	TF ± SD (mg RE/g)	T ± SD (mg CaE/g)	IC_50_ ± SD (μg/mL)
Crude extract	309.1 ^b^ ± 0.88	198.09 ^b^ ± 0.33	-	20.44 ^b^ ± 0.37
Dichloromethane	113.1 ^d^ ± 1.30	148.44 ^c^ ± 0.55	-	37.63 ^a^ ± 0.34
Ethyl acetate	522.4 ^a^ ± 1.12	220.48 ^a^ ± 0.30	3.86 ± 0.53	16.21 ^c^ ± 0.38
Butanolic	216.8 ^c^ ± 0.42	151.02 ^c^ ± 0.46	-	14.17 ^c^ ± 0.21
Ascorbic acid	-	-	-	14.86 ^c^ ± 0.28

Values are expressed as mean ± standard deviation. ^a–c^ Means with the different letters in each column are significantly different (*p* < 0.05) according to the Tukey test (n = 3).

No significant differences in the IC_50_ values of the ethyl acetate and butanolic fractions and those which showed the best results in the antioxidant capacity measured by the DPPH test. The most common methods to determine antioxidant activity in a practical, rapid and sensitive manner are those that involve a radical chromophore, simulating the reactive oxygen species, and the free radical DPPH, of purple coloration that absorbs at 515 nm, is one of the most widely used for *in vitro* evaluation of plant extracts and fractions. By the action of an antioxidant or a radical species, the DPPH is reduced to form diphenyl-picrylhydrazine (yellow), with a consequent decrease of the absorption [18]. This is in agreement with previous studies showing that these fractions are good sources of antioxidant compounds [17,19,20,21]. This fact can be explained on the basis of the similarity between compounds with high antioxidant activity extracted by these organic solvents. The ethyl acetate fraction showed a relatively high level of polyphenols, almost twice that of the butanolic fraction, yet it showed the same antioxidant capacity. A positive correlation was well established with the ethyl acetate fraction of *V. megapotamica* because the highest values of polyphenols and flavonoids in this fraction relate to the good DPPH radical inhibition results. This correlation between phenolics compounds and the antioxidant capacity (DPPH method) is in good agreement with the results of Mustafa *et al*. [22], Surveswaran *et al*. [23] and Janovik *et al*. [17]. However, some studies suggest that it is not always possible to correlate the total phenolics and antioxidant capacity [24,25]. In fact, the crude extract, which had the second highest content of total polyphenols, showed a higher IC_50_ than the butanolic fraction, whose total polyphenols value was lower. This can be explained by several factors, including the presence of different active compounds in the plant that can modify the antioxidant capacity, the synergistic effects of different compounds, the experimental conditions, and the mechanisms of the methods used for antioxidant reactions [26]. Structural factors include the nature of the phenolic groups and the changes caused by glycosylation [27]. There are also compounds that react strongly with the DPPH, and others that have a slower reaction rate [28]. 

The results for the content of condensed tannins in this study were not satisfactory, because only the ethyl acetate fraction gave a small amount of these compounds [3.86 ± 0.53 catechin equivalents (CaE)]. In a preliminary phytochemical analysis, the presence of tannins in the hydroalcoholic extract was revealed by reaction with ferric chloride [12]. According to Salminen *et al.* [29] several factors may be related to the low tannin content and the absence of solvent extractables in this study, such as seasonality and the influence of collection site, effect of air pollution and the effect of nutrient restriction soil. The extraction solvent and experimental methodology can also interfere, because according to the method it depends on the reaction of vanillin with vanillin tannins to form colored complexes. The success of this test depends on the type of solvent used, the concentration and nature of the acid, the reaction time, temperature and concentration of vanillin. 

### 2.2. DCFH-DA Method

**Figure 1:**
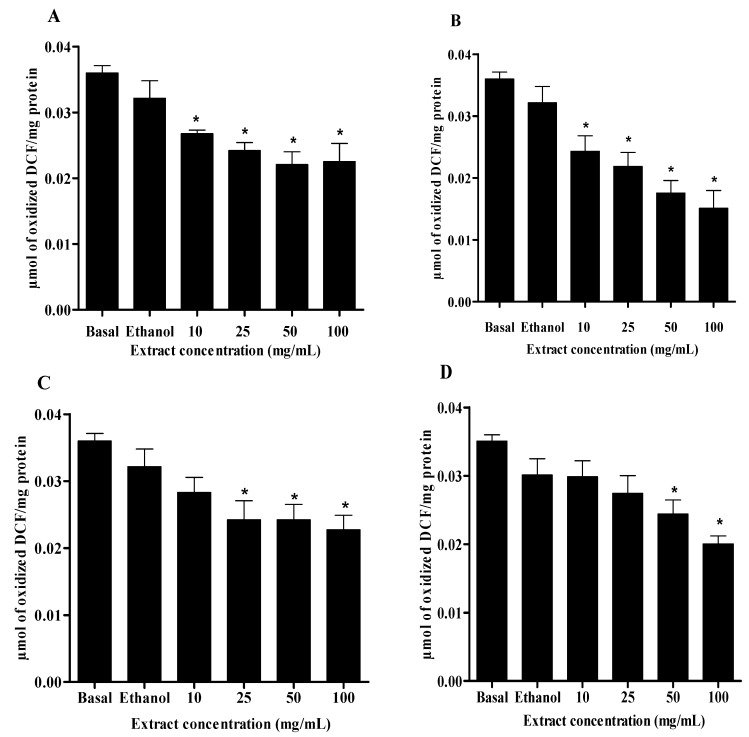
Effect of crude extract (**A**), dichloromethane (**B**), ethyl acetate (**C**) and butanolic fractions (**D**) of *V. megapotamica* leaves on scavenging of ROS in rat brain cells, by DCFH-DA method.

The crude extract and fractions were able to significantly reduce the oxidation of DCFH and consequently reduce the oxidative stress observed in rat brain cells, compared to the basal group, demonstrating pronounced antioxidant activity (Figure 1). The dichloromethane fraction and crude extract had a higher antioxidant capacity at all tested concentrations, already pronounced at the lower concentration tested (10 mg/mL). However, the ethyl acetate and butanolic fractions also showed good antioxidant activity, but at higher concentrations of 25 mg/mL and 50 mg/mL, respectively.

The fluorogenic compound DCFH-DA is a sensitive dye and has been utilized extensively as a marker for oxidative stress in cells [30]. It is known that once DCFH-DA enters the cell, the acetyl moiety is cleaved by intracellular esterase; subsequent oxidation by ROS, particularly H_2_O_2_ and hydroxyl radical, yields the fluorescent product, 2',7'-dichlorofluorescein (DCF) [31]. Thus, increases in DCFH oxidation to DCF (increases in DCF fluorescence) are suggestive of H_2_O_2_ or hydroxyl generation. Based on this, the principle of this assay is to evaluate the ability of antioxidants molecules in the extract to scavenge ROS produced by normal metabolism by cells, and then inhibit the oxidation of DCFH to DCF, observed by the decrease in fluorescence intensity. The results found in this study confirm that *V. megapotamica* leaves could exert a substantial effect against intracellular ROS formation.

Data are expressed as means ± S.D., (n = 3). * Significant difference from the basal group (*p* < 0.05).

### 2.3. Lipid Peroxidation

The results demonstrated the effect of different *V. megapotamica* fractions on TBARS production in brain, induced by Fe(II), since the brain tissue is vulnerable to free radical damage in view of the fact of its high consumption of oxygen and relatively low concentration of antioxidant enzymes and free radicals scavengers. 

**Figure 2:**
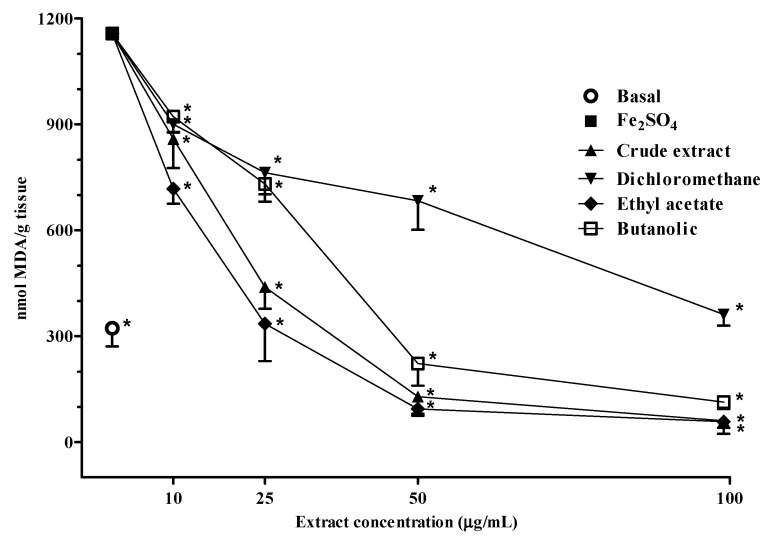
Effect of different concentrations of crude extract, dichloromethane, ethyl acetate and butanolic fractions from the leaves of *V. megapotamica* on Fe(II) (10 µM)-induced TBARS production in brain homogenates. Data are expressed as means ± S.D., (n = 3). Significant differences are indicated by * *p* ≤ 0.05 when compared with FeSO_4_ group.

In this assay, the IC_50_ values were in the following order: dichloromethane > butanolic > crude extract > ethyl acetate (Table 2 and Figure 2). The *V. megapotamica* fractions reduced the TBARS production considerably and this occurred in parallel with the increase of concentrations tested. All the fractions of the species under study decreased the MDA production at basal levels. However, the crude extract, ethyl acetate and butanolic fractions demonstrated this effect at lower concentrations when compared with the dichloromethane fraction. Ethanol was utilized in both fractions tested as the dilution vehicle and did not demonstrate any interference with the obtained results. Lipid peroxidation is an oxidative deterioration process of polyunsaturated fatty acids which are damaged by radical species. MDA is one of the most widely used biomarkers since it is one of the secondary products of lipid peroxidation [2,32]. These results suggested that the ethyl acetate fractions might contain the strongest lipid peroxidation inhibitory compounds, because is the fraction that has a 50% inhibition in the production of TBARS in a lower concentration. The inhibition of lipid peroxidation showed a positive relation with the total phenols levels. 

In a study moderate inhibition of the lipid peroxidation by ascorbic acid (IC_50_ = 117.81 ± 1.23 μg/mL) was found using a method similar to the performed in this work, demonstrating that the crude extract and fractions of *V. megapotamica* gave the best results [4]. Free Fe(II) can induce neurotoxicity and its levels are increased in some degenerative diseases [33]. The use of natural therapeutic antioxidant compounds can afford protection in a variety of *in vitro* and *in vivo* models of human pathologies, including neurotoxicity models [34,35].

**Table 2:** Lipid peroxidation (IC_50_/TBARS) for crude extract and fractions of *V. megapotamica.*

Crude extract/fractions	IC_50_ µg/mL(mean ± SD)
Crude extract	20.22 ± 4.27
Dichloromethane	72.72 ± 7.22
Ethyl acetate	16.36 ± 5.09
Butanolic	32.78 ± 3.06

SD: standard deviation.

### 2.4. Protein Carbonyl

**Figure 3:**
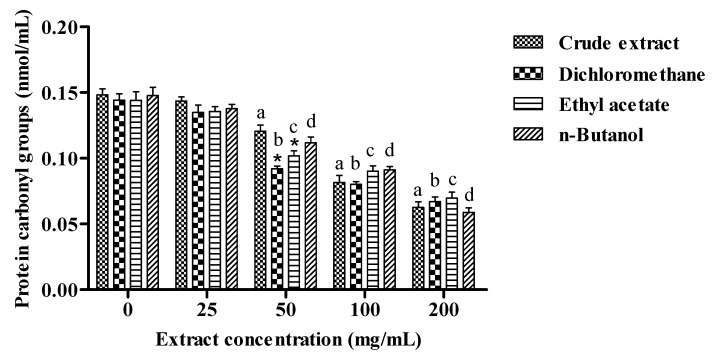
The effect of crude extract, dichlorometane, ethyl acetate and butanolic fractions of *V. megapotamica* on protein carbonyl groups production in plasma. Data are expressed as means ± SD (n = 4). The letters a, b, c, d means different from control (*p* < 0.05). * Different from crude extract (*p* < 0.05).

The study revealed a significant decrease in PCG levels in plasma, after incubation with the extracts at different concentrations (50, 100 and 200 mg/mL).The concentration of 25 mg/mL for all extracts was not able to reduce the PCG levels when compared to the control. These results showed a dose-dependent effect (Figure 3). This way was concluded that higher doses were more efficient than lower doses (25 mg/mL) since higher doses are richer in phenolic compounds. Furthermore, the dichloromethane and ethyl acetate fractions differ significantly when compared to the crude extract at a dose of 50 mg/mL, in reducing protein levels in plasma. The other doses did not show differences between the crude extract and fractions, demonstrating similar effects for all fractions at different concentrations. This result was expected, since the extracts of *V. megapotamica* showed considerable amounts of polyphenols which are responsible for the protective and antioxidant effects.

The action of reactive oxygen species on proteins has been widely demonstrated to increase the formation of carbonyl groups [36]. Moreover, carbonyl stress may be due to the damaging effect of various monodicarbonyls (such as MDA and HNE) and of hypochlorous acid (whose production is catalysed by myeloperoxidase in neutrophils) on proteins. However, in contrast to lipid peroxidation, protein oxidation does not have the feature of chain reactions. Also the level of carbonyl groups in circulating proteins is considered a useful marker of oxidative stress, which may have some advantages in comparison with the measurement of other parameters; in fact, these oxidation products are formed relatively early and are more stable. High levels of protein carbonyl groups (PCG) have been observed in several diseases, such as Alzheimer’s disease, rheumatoid arthritis, diabetes, sepsis, chronic renal failure, and in some malignancies [37]. 

A previous study demonstrated the inhibitory effects of four plants species—*Teucrium polium*, *Cyperus rotundus*, *Anethum graveolens* and *Nasturtium officinale*—against the formation of protein carbonyls, lipid peroxidation and reactive oxygen species. The authors attributed these protective effects of extract of each plant to its polyphenol content [38]. In another study, the hydroalcoholic extract of *Achillea santolina* proved to have marked antioxidant activity against protein oxidation in homogenate of liver rat [39].

### 2.5. HPLC-DAD Quantitative Analysis of Rosmarinic and Chlorogenic Acids (RA and CLA)

The crude extract and fractions from *V. megapotamica* were investigated for the presence of CLA and RA. In this study, was possible to quantify the RA in the leaves of *V. megapotamica* only in the ethyl acetate fraction (54.11 ± 0.30 mg/mL) and it was merely identified in the others fractions. In comparison with the RA, the CLA was found in much smaller amounts in the crude extract (0.87 ± 0.01), dichloromethane (0.74 ± 0.06) and *n*-butanol (1.23 ± 0.05) fractions. The presence of RA in high quantities can be closely related to the lowest values of IC_50_ obtained for ethyl acetate fraction in the DPPH and TBARS assays. 

A variety of biological activities has been described for RA. The main activities are antioxidant, anti-inflammatory, antibacterial and antiviral [40]. However the most important activity is its antioxidant capacity, which is attributed to the four hydroxyl groups present in the molecule that act as scavengers of radical species [41]. 

Previous works have demonstrated the preventive effects of RA and CLA against lipid peroxidation [42,43] and also in the strongest DPPH radical scavenging activity in different *in vitro* assays compared with other hydroxycinnamic acids [44]. In addition, was showed the antioxidant activity of RA in adsorbing and neutralizing free radicals, quenching singlet and triplet oxygen or decomposing peroxides [45]. The mechanism by which the phenolic acids exert their antioxidant activity is probably due to their chemical structures. The antioxidative activity of polyphenols is generally imputed to their hydroxyl groups, but it is not the only factor in determining the potency of their activities [44]. The capacity to inhibit hydroxyl radical is based mainly in the presence of *o*-dihydroxyl groups in aromatic rings, so caffeic, chlorogenic and rosmarinic acids possess stronger antioxidant activity than monophenolics, and also the presence of a carboxylic acid group [46,47].

Although CLA was found in lower concentrations in the extracts of *V. megapotamica*, this compound is found in most plant species [48] and a variety of studies have demonstrated the beneficial effects of CLA on different pathophysiological effects, such as antihypertensive and anti- hyperglycemic effects, prevention of the development of human colon cancer and inhibition of proliferation of tumor cells of different lines and anti-inflammatory action [49,50,51,52,53]. The phenolic acids found in this study for *V. megapotamica* are known to have many biological activities, which can thus be correlated with the popular use of this plant.

## 3. Experimental

### 3.1. Chemicals

All chemicals were of analytical grade. Solvents for the extractions, dichloromethane, ethyl acetate, ethanol and *n*-butanol were purchased from Merck (Darmstadt, Germany). Rutin, catechin, gallic, ascorbic, chlorogenic and rosmarinic acids, 2,2-diphenyl,1-picrylhydrazine (DPPH), dimethylsulfoxide (DMSO), tris-HCl, thiobarbituric acid and DCFH-DA were acquired from Sigma Chemical Co. (St. Louis, MO, USA). Folin-Ciocalteau reagent and iron sulfate (FeSO_4_) were acquired from Merck (Darmstadt, Germany). 

### 3.2. Animals

Male Wistar rats (3.0–3.5 months of age and weighing 270–320 g) were maintained in groups of 3–4 rats per cage. They had continuous access to food and water in a room with controlled temperature (22 ± 3 °C) and on a 12-h light/dark cycle with lights on at 7:00 a.m. The animals were maintained and used in accordance to the guidelines of the Brazilian Association for Laboratory Animal Science (COBEA) (project number 23081.005770/2009-38). 

### 3.3. Plant Collection and Extractions

Leaves of *V. megapotamica* were collected in Santa Maria (Rio Grande do Sul State of Brazil) in December of 2010. A dried voucher specimen is preserved in the herbarium of the Department of Biology at Federal University of Santa Maria by register number SMBD 13071. Plant material was dried at room temperature and powdered in a knife mill. The leaves (1,272.68 g) were macerated at room temperature with 70% ethanol (6.360 mL) for a week with daily shaking; the solvent was renewed several times. After filtration, the hydroalcoholic extract was evaporated under reduced pressure at a temperature below 40 °C, in order to obtain the aqueous extract; part of this aqueous extract was evaporated to dryness to furnish a crude extract. The remaining aqueous extract was partitioned with solvents of increasing polarity: dichloromethane, ethyl acetate and *n*-butanol. Finally, the fractions obtained were concentrated to dryness on a rotary evaporator.

### 3.4. Determination of Total Phenolics

Total phenolic contents were measured using Folin–Ciocalteu method, slightly modified as described by Boligon *et al.* [1]. Gallic acid was used as standard and samples were read in triplicate at 730 nm in a spectrophotometer. Total phenolic content was expressed in milligrams equivalents of gallic acid (GAE) per gram of each fraction. The equation obtained for the calibration curve of gallic acid was Y = 15.635x + 0.0194 (r = 0.9671).

### 3.5. Determination of Total Flavonoids

Total flavonoids were determined according to a slightly modified colorimetric method described by Woisky and Salatino [54]. Samples were prepared at a concentration of 1.0 mg/mL in methanol. Briefly, each sample (0.5 mL) was added to AlCl_3_ (2%, w/v, 0.5 mL) and methanol (2.5 mL). The absorbances were measured at 420 nm against the blank. Rutin was used as standard and samples were performed in triplicate. The flavonoid content was established in milligrams equivalents of rutin per gram of each fraction. The equation obtained for the calibration curve of rutin was y = 4.2561x + 0.0052 (r = 0.9999). 

### 3.6. Determination of Condensed Tannins

Condensed tannins were determined by the vanillin method according to Morrison *et al.* [55]. Samples were prepared at a concentration of 25.0 mg/mL in methanol. To each sample (0.1 mL) were added methanol (0.9 mL), solution A (8.0 mL concentrated HCl in 100.0 mL methanol, 2.5 mL) and solution B (1.0 g vanillin in 100.0 mL methanol, 2.5 mL). The absorbances were read at 500 nm against the blank of each sample. Catechin was used to make the calibration curve and the test was performed in triplicate. The condensed tannins content was expressed in milligram equivalents of catechin per gram of each fraction. The equation obtained for the calibration curve of catechin was y = 0.0015x + 0.0005 (r = 0.9936).

### 3.7. Radical—Scavenging Activity-DPPH Assay

The antioxidant activity of the fractions and the crude extracts was evaluated by monitoring its ability in quenching the stable free radical DPPH, according to a slightly modified method previously described by Choi *et al.* [56]. The DPPH quenching ability was expressed as IC_50_ (the extract concentration required to inhibit 50% of the DPPH in the assay medium). Six different ethanol dilutions of each sample at 250, 125, 62.5, 31.25, 15.62, and 7.81 µg/mL were tested. A solution of DPPH (1 mL; 0.3 mM) in ethanol (2.5 mL) was used as a negative control and ascorbic acid in the same concentrations used for the samples provided the positive control. The absorption was measured at 518 nm and ethanol was used to calibrate the spectrophotometer. The test was performed in triplicate and the antioxidant activity was calculated by the equation:

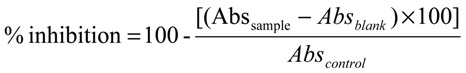
(1)
where Abs_sample_ is absorbance of each fraction; Abs_blank_ is absorbance of fractions without adding the DPPH; Abs_control_ is absorbance the solution of negative control.

### 3.8. DCFH-DA Method

The substrate DCFH-DA was utilized to measured intracellular formation of ROS, according to Myrhe *et al.* [57]. The supernatant of rat brain homogenate was incubating at 37 °C with different concentrations of crude extract and fractions of *V. megapotamica*. After 1 hour, aliquots were removed and DCFH-DA (5 µM) was added to the medium and incubation continued for 1 hour in the dark. The fluorescence was measured using 488 nm for excitation and 520 nm for emission. ROS levels (expressed as µmol of oxidized DCF per mg protein) were calculated by interpolation in a standard curve of oxidized DCF (constructed in parallel), corrected by the content of protein [58]. Ethanol was used as negative control. The assay was performed in triplicate and data were expressed as mean ± SD.

### 3.9. *In Vitro* Fe (II)-Induced Lipid Peroxidation in Rat Brain

Rats were killed and the encephalic tissue was rapidly dissected and placed on ice. Tissues were immediately homogenized in cold 10 mM Tris-HCl, pH 7.5 (1/10, w/v). The homogenate was centrifuged for 10 min at 4,000 × *g* to yield a pellet that was discarded and a low-speed supernatant (S1) that was used for the TBARS assay. An aliquot of 100 μL of S1 was incubated for 1 h at 37 °C with freshly prepared FeSO_4_ (10 μM), in the presence of different fractions of *V. megapotamica*. Then, TBARS production was determined as described by Ohkawa *et al.* [59]. 

### 3.10. Protein Carbonyl Groups

The plasma content of protein carbonyl groups was evaluated with the Levine method [60]. The crude extract and fractions were diluted in Phosphate-buffered saline (PBS) buffer in the desired concentrations. In a test tube, was placed plasma (1 mL) and extract diluted in PBS buffer (1 mL). Briefly, plasma (100 µL) in the absence or presence of crude extract and fractions in different dilutions was incubated with a 20 mM 2,4-dinitro-phenylhydrazine (DNPH) solution (100 µL) for 60 min. The proteins were precipitated from the solution with the use of 20% trichloroacetate; the protein pellet was washed three times with ethanol and ethyl acetate and resuspended in 1 mL of 6 M guanidine at 37 °C for 15 min. The carbonyl content was determined by reaction with DNPH and the absorbances were read at 366 nm (molar absorption coefficient, 22.000 M^−1^/cm) [37]. The determination of total protein in plasma was conducted using a commercial Labtest^®^ kit as recommended by the manufacturer. All tests were performed in triplicate, and the carbonyl content was expressed as nmol/g protein.

### 3.11. HPLC-DAD Quantitative Analysis of Rosmarinic and Chlorogenic Acids

High performance liquid chromatography was performed with a Prominence Auto-Sampler (SIL-20A) equipped with Shimadzu LC-20AT (Shimadzu, Kyoto, Japan) reciprocating pumps connected to a DGU-20A5 degasser and CBM-20A integrator. UV-VIS detector DAD SPD-M20A and Software LC solution 1.22 SP1 were used. Reverse phase chromatography analyses were carried out with a Phenomenex C-18 column (4.6 mm × 250 mm) packed with 5 µm diameter particles, volume injection was 40 µL and the gradient elution was conducted according to the Evaristo and Leitão [61] method with minor modifications. Mobile phase consists of water containing 2.0% acetic acid (solvent A) and methanol (solvent B). The UV absorption spectra of the standards as well as the samples were recorded in the range of 230–400 nm. Stock solutions of standards were prepared in methanol in the range of 0.0025–0.045 mg/mL. Samples and standards solutions as well as the mobile phase were degassed and filtered through 0.45 µm membrane filter (Millipore). Chromatographic operations were carried out at ambient temperature and in triplicate. Identification of the compounds was done by comparison of their retention’s time and UV absorption spectrum with those of the standards.

### 3.12. Statistical Analysis

The results obtained for DPPH, total phenolics, flavonoids, tannins assays were analyzed statistically by one-way analysis of variance (ANOVA), followed by Tukey test using the statistical package SAS (2001). Data from the TBARS, protein carbonyl assay and scavenging of ROS by DCFH-DA method were analyzed statistically by one-way analysis of variance (ANOVA), followed by Duncan’s multiple range tests when appropriated using the statistical software SPSS 10.0 for Windows, and *p* < 0.05, *p* < 0.01, or *p* < 0.001 when appropriate were considered significant. Data were expressed as mean ± SD.

## 4. Conclusions

The fractions and crude extract of *V. megapotamica* tested here have effective antioxidant capacity, being the ethyl acetate fraction the one with the strongest lipid inhibitory actitity and the highest contents of phenolics compounds, flavonoids and tannins. HPLC/DAD analysis performed with *V. megapotamica* revealed the presence of two phenolic compounds: chlorogenic and rosmarinic acid, and the ethyl acetate fraction showed the larger number of phenolics. Higher dosages of the extracts were more effective in reducing levels of plasma proteins carbonyl and also shown to be able to remove reactive species by DCFH-DA method, reducing oxidative stress in all tested factions. Considering that natural substances can be responsible for the protective effect against the risk of many disease processes, the results described in this paper suggest that *V. megapotamica* has important antioxidant activity, which may be correlated to its popular use, serving as a stimulus for further study to assess the antioxidant activity of compounds isolated from the leaves of this species.
